# Regulation of *Mariner* Transposition: The Peculiar Case of *Mos1*

**DOI:** 10.1371/journal.pone.0043365

**Published:** 2012-08-14

**Authors:** Jérôme Jaillet, Murielle Genty, Jeanne Cambefort, Jacques-Deric Rouault, Corinne Augé-Gouillou

**Affiliations:** 1 Innovation Moléculaire Thérapeutique, EA 6306 – Université François Rabelais, Parc Grandmont, Tours, France; 2 Laboratoire Evolution, Génomes et Spéciation – CNRS UPR9034, Gif-sur-Yvette, France; 3 Université Paris-Sud 11, Orsay, France; University of Poitiers, France

## Abstract

**Background:**

*Mariner* elements represent the most successful family of autonomous DNA transposons, being present in various plant and animal genomes, including humans. The introduction and co-evolution of *mariners* within host genomes imply a strict regulation of the transposon activity. Biochemical data accumulated during the past decade have led to a convergent picture of the transposition cycle of *mariner* elements, suggesting that *mariner* transposition does not rely on host-specific factors. This model does not account for differences of transposition efficiency in human cells between *mariners.* We thus wondered whether apparent similarities in transposition cycle could hide differences in the intrinsic parameters that control *mariner* transposition.

**Principal Findings:**

We find that *Mos1* transposase concentrations in excess to the *Mos1* ends prevent the paired-end complex assembly. However, we observe that *Mos1* transposition is not impaired by transposase high concentration, dismissing the idea that transposase over production plays an obligatory role in the down-regulation of *mariner* transposition. Our main finding is that the paired-end complex is formed in a cooperative way, regardless of the transposase concentration. We also show that an element framed by two identical ITRs (Inverted Terminal Repeats) is more efficient in driving transposition than an element framed by two different ITRs (*i.e.* the natural *Mos1* copy), the latter being more sensitive to transposase concentration variations. Finally, we show that the current *Mos1* ITRs correspond to the ancestral ones.

**Conclusions:**

We provide new insights on intrinsic properties supporting the self-regulation of the *Mos1* element. These properties (transposase specific activity, aggregation, ITR sequences, transposase concentration/transposon copy number ratio…) could have played a role in the dynamics of host-genomes invasion by *Mos1*, accounting (at least in part) for the current low copy number of *Mos1* within host genomes.

## Introduction

Transposable elements make up the largest fraction of many eukaryotic genomes [1]. Among them, *mariner* elements represent one of the most widespread groups of DNA transposons. *Mariners* have successfully colonized various genomes, especially animal genomes. They constitute an ancient transposon family, and have been assigned to various subfamilies (*briggsae*, *cecropia*, *elegans*, *irritans*, *mauritiana*, *mellifera*…). Several thousands *mariner* sequences can be found in genome databases. According to Rouault *et* *al* [2], these subfamilies have been further divided into tribes, which are groups of highly related elements derived from a common ancestor or founder. The *mariner* elements life cycle depends on frequent horizontal transfer into new hosts. A general model of this life cycle [3]–[5] underscores the initial invasion of the germ line of an organism by a single founder element, which then increases its copy number and spreads through the population via sexual reproduction. As the copy number increases, the rate of transposition slows down by either intrinsic, emergent, or host-mediated regulatory mechanisms. Random mutations accumulate, and inactivate most – or all – copies. Some elements can escape this fate through horizontal transfer, and establish a novel lineage in new populations and/or species.

**Figure 1:**
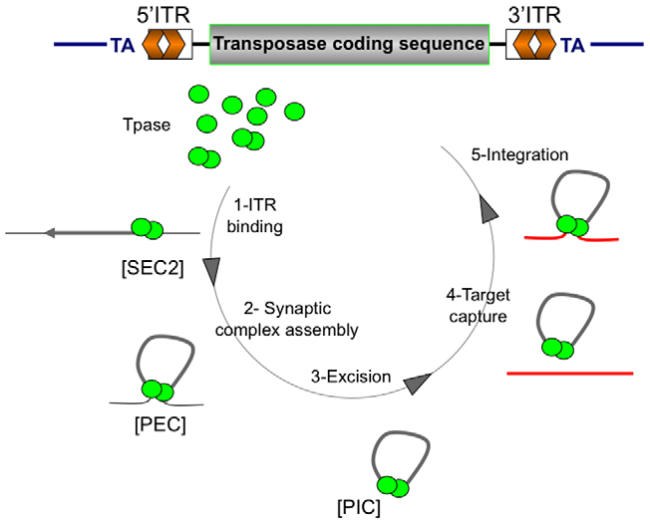
*Mariner* transposition cycle. A representative *mariner* element is depicted at the top of the figure, with its main components, *i.e.* the transposase coding sequence (in grey), the inverted terminal repeats (5′ITR and 3′ITR, in orange), and the TA dinucleotide flanking the element (landmark for transposition). According to published data [7] [18], *Mos1* transposition consists of five main steps: (**1**) dimerization of MOS1 proteins, the *Mos1* transposase (green circle) for subsequent ITR binding, thus forming SEC2 (Single-end complex 2). (**2**) Synaptic complex assembly is obtained by the addition of the second ITR to SEC2, thus forming the PEC (Paired-end complex). (**3**) DNA strands are then cleaved by the transposase, promoting the excision. Once the PIC (Pre-integration complex) has been produced, the capture of the target DNA occurs (**4**), followed by the integration of the element into a TA target dinucleotide (**5**). The results presented in this study argue for generalized this model to all *mariner* elements.

Biochemical data accumulated during the past decade from three models (*Mos1* from *D. mauritiana*, *Himar1* from *H. irritans*, and *Hsmar1* from *H. sapiens*) have led to a convergent picture of the transposition cycle of *mariner* elements. All *mariners* are short elements, about 1300 base pairs, flanked by inverted terminal repeats (ITRs). They transpose using a cut and paste mechanism. *In vitro*, the only actors required for the transposition are the transposase and the ITRs (Figure 1) [6]–[8]. When transposition occurs *in vivo*, survival of the cell requires the repair of the excision locus by host factors. The transposition model established in vitro is in agreement with the diversity of host genomes successfully colonized by mariner elements, and suggests that *mariner* transposition does not rely on specific host factors. However, this model can not explain why *Mos1* is the least efficient *mariner* to transpose in mammal cells [9]–[10]. This led us to wonder whether an apparent similarity in transposition cycle might hide differences in the intrinsic parameters that control transposition. The first and obvious parameter is that *Mos1*-related elements have two different (5′ and 3′) ITRs whereas the two others (*Himar1*, and *Hsmar1*) have identical ITRs. Some other biochemical parameters controlling *Mos1* transposition (transposase amount, ITR sequences) have been investigated using different approaches (e.g. genetics or biochemical) [11]–[13], leading to a puzzling picture of *Mos1* transposition regulation. Considering that the mechanisms driving transposable elements auto-regulation is a key concern for both population geneticists and scientists involved in transposon-tools development, we have reinvestigated the intrinsic parameters that control *mariner* transposition.

Using *in vitro* approaches, we checked whether variation in copy number and transposase concentration can mediate *Mos1* transposition regulation. We investigated the importance of the transposase (MOS1) concentration in the paired-end complex (PEC) assembly (an early stage in *mariner* transposition, Figure 1). We observed that an excess of MOS1 prevents PEC assembly. In contrast, *Mos1* transposition was not sensitive to high transposase concentrations, dismissing the idea that the transposase over production plays an obligatory role in the down-regulation of *mariner* transposition, at least *in vitro.* Taken together, our data suggested that the PEC is formed in a cooperative way, regardless of transposase concentration. In addition, we found that a transposon framed by two identical ITRs, having both high affinity binding-sites for the transposase, was about 20 times more efficient in driving transposition than an element framed by two different ITRs (*i.e.* the natural *Mos1* element), the latter being more sensitive to transposase concentration variations. Our results revealed differences in the intrinsic parameters controlling the transposition of different *mariner* elements and improved our understanding of the dynamics of *mariners* transposition.

## Results

### Effect of transposase concentration on paired-end complexes (PEC) assembly

The first description of a PEC involving the *Mos1* transposase was reported a decade ago [14]. This complex was shown to contain two ITRs plus an undefined number of transposase molecules, and was not detected in non-catalytic conditions (*i.e.* without MgCl_2_). We subsequently showed that *Mos1* transposition was initiated by the binding of one transposase dimer at one ITR [15]. By analogy with Class-II elements transposition mechanisms [16], we proposed that *Mos1* transposition required a synaptic complex containing two ITRs and four transposase [17]. More recently, this hypothesis was ruled out by the elucidation of the crystal structure of the *Mos1* PEC [18] showing two ITRs and two transposase molecules in one PEC.

**Figure 2:**
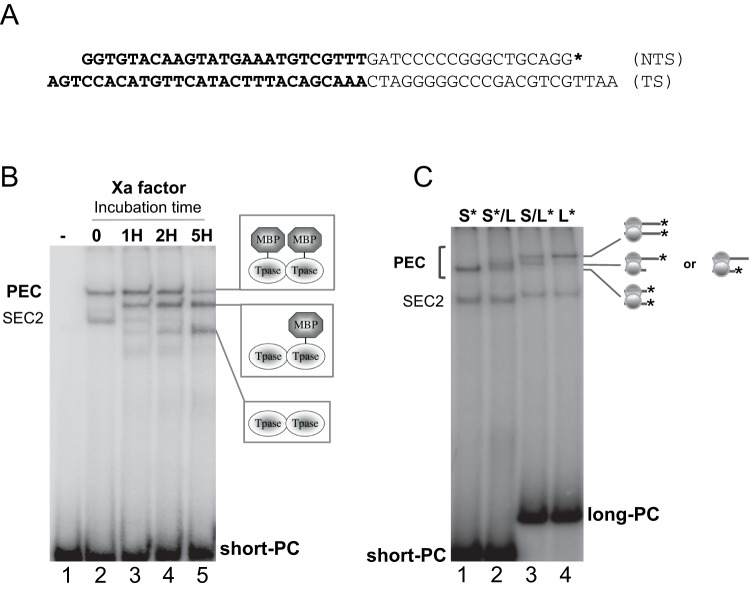
The PEC contains two ITRs and two transposases. A. Sequence of the double-strand oligonucleotide (short-PC: short pre-cleaved ITR) used in EMSAs. TS: transferred strand; NTS: non-transferred strand. The 3′ITR is shown in bold, with a 3-bases overhang in 3′ of the TS [14]. Inner sequence: narrow letters. The long-PC oligonucleotide (long pre-cleaved ITR) has the same sequence, with a longer inner sequence. An asterisk marks the position of the ^32^P labeling. **B.** Short/long transposase analyses. EMSAs were performed with 250 nM of short-PC (short pre-cleaved) labeled ITR (as a probe), and 250 nM of purified MBP-MOS1. Lane 1: probe alone, Lane 2: complexes assembly without factor-Xa treatment. Lanes 3 to 5, complexes were subjected to factor-Xa cleavage (1H, 2H and 5H respectively) before electrophoresis. The MOS1 dimer in the PEC was assayed here. We have taken advantage of the fact that the MBP-MOS1 fusion protein contains a cleavage site for factor-Xa between MBP and MOS1. If the PEC contains MBP-MOS1 dimer, then a three-band pattern is expected after cleavage of factor-Xa: one band containing uncleaved MBP-MOS1 (in native PEC, as seen at T = 0), one band containing one cleaved and one uncleaved MOS1 in the complex, and one band containing two cleaved MOS1s in the complex. SEC2 disappears as a result of the factor-Xa cleavage, since it contains two MBP-MOS1 molecules that are converted into MOS1 molecules by the release of the MBP moiety, giving bands with faster mobility in electrophoresis. The proteins present in the various PECs are drawn on the right. **C.** Short/long ITR analyses. EMSAs were performed with 250 nM of purified MBP-MOS1 and 250 nM of short/long ITR combinations (as indicated). The number of ITRs in the PEC is assayed here. The ITRs present in the complexes are drawn on the right. Short-PC: short pre-cleaved ITR. Long-PC: long pre-cleaved ITR. S*: labeled short-PC. L*: labeled long-PC.

In the present study, PEC assembly was performed using pre-cleaved *Mos1* 3′ITRs (to bypass the requirement for catalytic conditions) and purified *Mos1* transposase at an equimolar ratio. MOS1 was produced as a fusion protein linked to the maltose-binding protein (MBP-MOS1), which is used as a tag in the purification process. The presence of an MBP tag has been shown to have no influence on the enzymatic properties of the transposase (ref.) According to the accepted nomenclature, the complex expected in these conditions is not strictly a PEC (which designates a pre-cleavage paired end complex), but a PIC (a cleaved pre-integration complex). Since this is the complex whose crystal structure has been published recently, we have chosen to keep the nomenclature given by the authors, and designated this complex “PEC" throughout the manuscript. The stoechiometry of complexes formed using pre-cleaved *Mos1* 3′ITR was assayed by mobility shift assays (EMSAs) (Figure 2). In standard conditions (Figure 2B, lane 2 & 2C, lane 1), two complexes were detected. The one having a faster mobility in EMSA had been previously identified as a single-end complex that contains a transposase dimer [7], and further analyses confirmed its stoechiometry. For convenience, this complex is designated “SEC2" despite the fact that this acronym usually refers to a pre-cleavage single-end complex. The second complex migrates more slowly. Herein, we demonstrate that this complex is indeed a PEC.

The presence of a MOS1 dimer in the PEC was assayed by EMSA. We took advantage of the cleavage site for the factor-Xa protease between the MBP and MOS1 moieties in MBP-MOS1 fusion protein. If the PEC contains an MBP-MOS1 dimer, three bands would be expected following factor-Xa cleavage: one band containing uncleaved MBP-MOS1, one containing both a cleaved and an uncleaved MOS1 monomer, and one band containing two cleaved MOS1s. Our data demonstrate that the PEC does contain two transposase monomers since it satisfies the conditions expected following factor-Xa cleavage (Figure 2B). On the other hand, “full-length" SEC2 disappears as a result of the factor-Xa cleavage. SEC2 contains two MBP-MOS1 molecules that are converted into MOS1 molecules by the release of the MBP moiety, giving bands migrating faster in electrophoresis.

We prepared samples containing a mixture of long and short ITR DNAs to assay the number of ITRs in the PEC. This method is well established to determine whether a complex contains one or a pair of transposon ends [19]. If an ITR/transposase complex contains two ITRs, a reaction containing a short, labeled ITR (S*) and a long, unlabeled ITR (L) will produce two visible complexes in EMSA: one faster migrating complex with two short labeled ITRs (S*/S*), and one slower migrating complex with a short labeled ITR, and a long, unlabeled ITR (S*/L). The complex containing two long unlabeled ITRs (L/L) will be undetectable. A two bands pattern is also expected in the reciprocal experiment using one short, unlabeled (S) and one long, labeled ITR (L*). Control experiments were done using either the short-labeled ITR (S*) or the long labeled one (L*) alone. Our results showed that the PEC contained two ITRs (Figure 2C). As expected, SEC2 was insensitive to the presence of long and short ITR mixtures as it contained a single ITR.

**Figure 3:**
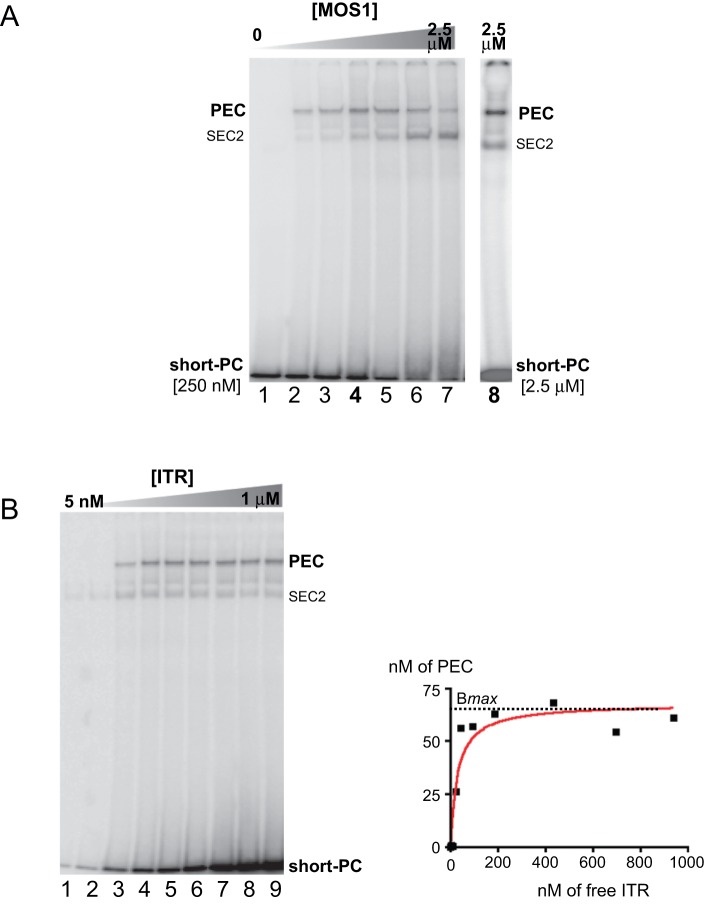
The PEC is sensitive to transposase concentration in EMSAs. A. Relationships between PEC assembly and MOS1 concentration. EMSAs were performed with 250 nM of short-PC labeled ITR, excepted in lane 8, and various amount of purified MBP-MOS1, ranging from 0 to 2.5 µM. Lane 1: no MOS1, lane 2: 50 nM, lane 3: 100 nM, lane 4: 250 nM, lane 5: 500 nM, lane 6: 1 µM, lane 7: 2.5 µM. Lane 8: 2.5 µM of short-PC and 2.5 µM MOS1. Short-PC: short pre-cleaved ITR. **B.** Relationships between PEC assembly and ITR concentration. *Left panel*: EMSAs were performed with various amount of short-PC labeled ITR, and 100 nM of MOS1. Lane 1: 5 nM ITR, lane 2: 10 nM, lane 3: 50 nM, lane 4: 100 nM, lane 5: 150 nM, lane 6: 250 nM, lane 7: 500 nM, lane 8: 750 nM, lane 9: 1 µM. *Right panel*: the amount of ITR in the PEC (nM) was plotted against the concentration of free ITR (nM). The maximum amount of bound ITR obtainable for MOS1 concentration of 100 nM, B*max*, is indicated (dotted line). Short-PC: short pre-cleaved ITR.

Our results showed that the complex of lower mobility contains two ITRs and two transposases. Thus, this complex satisfies to the stoechiometry of a PEC, in agreement with the crystal structure [18]. The impact of MOS1 concentrations on PEC assembly was then addressed using a fixed ITR concentration (250 nM) and various MOS1 concentrations ranging from 0 to 2.5 µM (Figure 3A). Our data showed that the PEC was formed at low concentrations of transposase (50 nM, Figure 3A-lane 2). The amount of PEC increased with the transposase concentration, reaching a maximum with 500 nM of transposase (Figure 3A-lane 5). However, we noted that part of the labeled ITR remained unbound, suggesting that the purified transposase was unlikely to be 100% active. This was confirmed by the results presented in Figure 3B. The presence of higher MOS1 concentrations hindered PEC assembly, and favoured the formation of SEC2 (Figure 3A-lanes 6,7). This observation suggested the titration of the ITR by the transposase: at high transposase concentration, no free ITR was available to allow PEC assembly. An alternative explanation is that increasing transposase concentration leads to inactive protein-protein interactions between MOS1 monomers. The inactive dimers could still bind DNA but not support the next step of the canonical transposition reaction, namely the PEC formation. This explanation was ruled out by an experiment in which high transposase concentration (2.5 µM) was mixed with high ITR concentration (2.5 µM). The observed PEC assembly (Figure 3A-lane 8) supported the ITR titration hypothesis.

As previously described, the fraction of active transposase in the purified sample was addressed in EMSA using a fixed transposase concentration (100 nM) and various ITR concentrations ranging from 0 to 1 µMm (Figure 3B-right panel). For each condition, the amount of free ITR (in nM) was plotted against the amount of ITR (in nM) engaged in the PEC (Figure 3B-left panel). The resulting graph allowed the evaluation of B*max*, *i.e.* the maximum amount of bound ITR obtainable for MOS1 concentration of 100 nM. Since the PEC contained an equimolar amount of ITRs and transposase, B*max* gives a direct measurement of the proportion of the active transposase in the purified sample. We obtained a B*max* of ≈65 nM (+/−8%, r^2^ = 0.93), suggesting that about 2/3 of the transposase was active in our purified sample. These data are in agreement with the observations made in Figure 3A.

### Effect of transposase and transposon concentration on *in vitro* transposition assays

**Figure 4:**
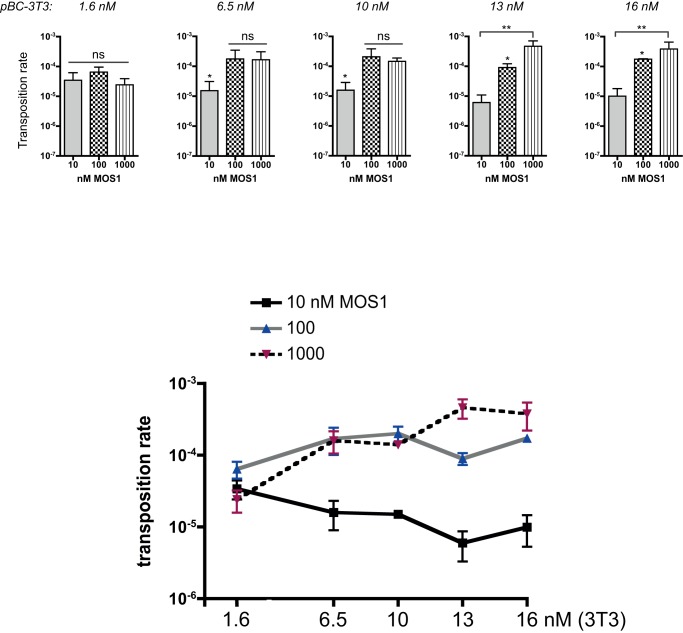
pBC-3T3 transposition rates. *Top panels*: Transposition rates were assayed with various amounts of pBC-3T3 (1.6 to 16 nM) and three MOS1 concentrations: 10 nM (grey bars), 100 nM (checkerboard bars), 1 µM (hatched bars). Each bar is the mean (+/− SD) of at least five independent assays. Kruskal-Wallis and post hoc tests were used to monitor the significance of the differences. ns: no statistic differences. (*) p<0.05. (**) p<0.005. *Bottom panel*: Transposition rates (from the top panel) are plotted as a function of the amount of pBC-3T3 used in the assay, for each transposase concentration. 10 nM MOS1: black line, 100 nM: gray line, 1 µM: dotted line.

The results of EMSA experiments showed that transposase over-concentrations (relative to that of the ITRs) prevented PEC assembly. If this was true, it should also prevent transposition, in an experiment mimicking the entire transposition cycle. We tested the effect of MOS1 concentration using an *in vitro* genetic “hop" experiment, which is the most sensitive test available as yet. Since our previous results have suggested that the transposase/ITR ratio is a crucial parameter, the assays were set using various amount of pseudo-*Mos1*. Purified MBP-MOS1 transposase was incubated with the pBC-3T3 plasmid, which was used both as the transposon donor and the target plasmid. This plasmid contains the pBR322 tetracycline resistance gene (without promoter) framed by two identical *Mos1* ITRs (corresponding to the 3′ITR sequence). This reconstitutes a pseudo-*Mos1* element named 3T3. pBC-3T3 is unable to confer tetracycline resistance to *Escherichia coli* cells at concentrations over 10 mg/ml. We took advantage of the fact that the *cat* gene (present in the pBC backbone) is a hotspot for *Mos1* integration [20]–[21]. Consequently, transposition events were revealed by promoter tagging, the tetracycline resistance being activated through the *cat* gene promoter. Transposition events were recovered by bacterial transformation with selection for tetracycline resistance, as a gain-of-function landmark for transposition [22]. We tested three MOS1 concentrations (10, 100 nM and 1 µM) and various pBC-3T3 amounts (1.6, 6.5, 10, 13, and 16 nM). Each condition was assayed at least five times. Our data (Figure 4-top) call for several comments. Transposition rates recovered with low pBC-3T3 amounts (1.6 nM of 3T3) were insensitive to transposase concentration, since Kruskal-Wallis tests gave no significant difference between the transposition rate values, whatever the transposase concentration. This suggested that the transposon concentration was the only limiting factor of the reaction. However, those data were different from those expected from the EMSA results (Figure 3A), which predicted that the transposition rate would decrease with increasing transposase concentrations, as ITR saturation occurs. In agreement with preliminary results about *Mos1* [23], our transposition data might indicate that ITR saturation did not occur during the assay. The ITRs were not likely to behave the same in EMSA and in transposition assays, changing the way by which the second ITR is added to SEC2 to form the PEC. In EMSA, the PEC assembly depends mainly on stochastic interactions between SEC2 and free remaining ITRs. In transposition assays, the recruitment of the second ITR may occur cooperatively, taking benefit from the negative supercoiling of the plasmid DNA, which places the second ITRs close to the preformed SEC2.

For moderate amounts of pBC-3T3 (6.5 and 10 nM of 3T3), the transposition rate was significantly higher for 100 and 1000 nM than for 10 nM transposase, suggesting that 10 nM was a limiting transposase concentration for promoting the transposition of 6.5 to 10 nM of 3T3. This result was consistent with the fraction of active purified transposase being only about 65%. The same reasoning applies for larger amount of pBC-3T3 (13 and 16 nM) for which the transposition rates increased with the transposase concentration.

To further analyze relationships between transposition rates, transposase concentration and transposon amounts, transposition rates were plotted as a function of the amount of plasmid, for each transposase concentration (Figure 4-bottom). For a low transposase concentration (10 nM), the increase in 3T3 copies number did not lead to an increase in transposition rate, the transposase being the limiting factor. For higher transposase concentrations, an increase in 3T3 copy number leaded to an increase in transposition rate until the available transposase becomes limiting. Transposition inhibition (*i.e.* a decrease in transposition rate) at high transposase concentration was never observed. This confirmed that two parameters account for transposition efficiency: the transposase concentration and the transposon amount.

**Figure 5:**
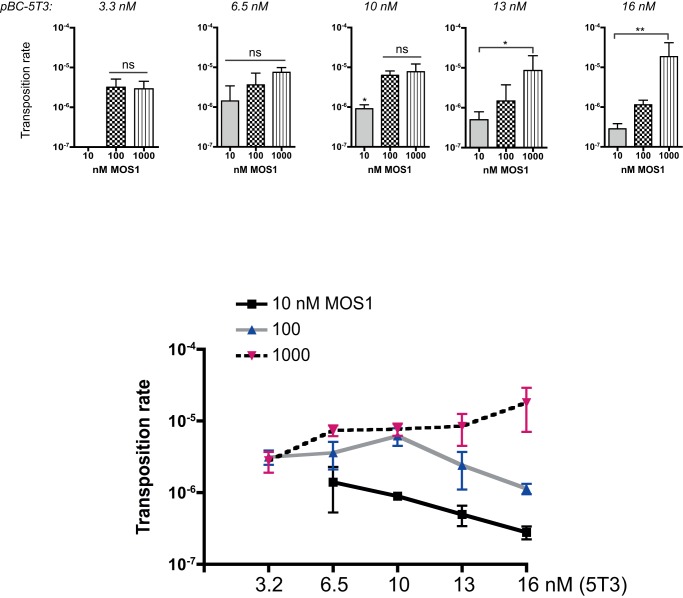
pBC-5T3 transposition rates. *Top panels*: Transposition rates were assayed with various amounts of pBC-5T3 (3.2 to 16 nM) and three MOS1 concentrations: 10 nM (grey bars), 100 nM (checkerboard bars), 1 µM (hatched bars). Each bar is the mean (+/− SD) of at least five independent assays. Kruskal-Wallis and post hoc tests were used to monitor the significance of the differences. ns: no statistic differences. (*) p<0.05. (**) p<0.005. *Bottom panel*: Transposition rates (from the top panel) are plotted as a function of the amount of pBC-5T3 used in the assay, for each transposase concentration. 10 nM MOS1: black line, 100 nM: gray line, 1 µM: dotted line.

The same analyses were made using the pBC-5T3 plasmid (which contains the two different *Mos1* natural ITRs) as the transposon donor and the target plasmid. The only difference was the use of 3.2 nM of pBC-5T3 as the lowest plasmid amount (instead of 1.6 nM for the pBC-3T3) due to lower efficiency of transposition with pBC-5T3. This feature is illustrated by the lack of detectable transposition events for 3.2 nM of pBC-5T3 and 10 nM transposase (Figure 5-top). Moreover, the maximum transposition rates observed were 5.10^−4^ for the 3T3 (1 µM transposase and 16 nM of pBC-3T3) and 2.10^−5^ for the 5T3 (at identical concentrations). Results were very similar to those obtained with the 3T3 construct, with only slight differences. For assays involving 6.5 nM of pBC-5T3, Kruskal-Wallis tests gave no significant difference between the transposition rates, whatever the transposase concentration. This suggested that 6.5 nM of 5T3 was the limiting transposon concentration, which was not the case for the 3T3. The fact that 5T3 contains a single high affinity binding-site for the transposase (a single 3′ITR) might alter the sensitivity threshold of transposase concentration. Data obtained using 10, 13 and 16 nM of pBC-5T3 were about the same as those obtained with the pBC-3T3, except for the transposition rates, which were lower. The same trend observed for the 3T3 (*i.e.* reaching a plateau of transposition rate when the 3T3 copies number increased at a constant and low transposase concentration, 10 nM) was observed in Figure 5 (bottom). It seems to be also the case at a moderate transposase concentration (100 nM). Hence, concerning 5T3, which is closer to the “natural" transposon structure, our results argue for a mechanism that goes beyond the limiting amount of transposase in the regulation of *Mos1* dynamics. In fact, our results emphasize the importance of the ratio of transposase level over the transposon copy number, which varies between 3T3 and 5T3.

### ITR sequences and the regulation of transposition: a clue for *mariner* evolution?

The results reported in the previous section showed that the transposition was more efficient when the pseudo-transposon bear two identical 3′ITR (corresponding to the 3T3) than the natural *Mos1* 5′ITR and 3′ITR (corresponding to the 5T3). The low amplification of *Mos1* in natural population [24] could rely (at least in part) on this feature. Therefore, we checked whether the ancestral *Mos1* copy already contained divergent ITRs.

**Figure 6:**
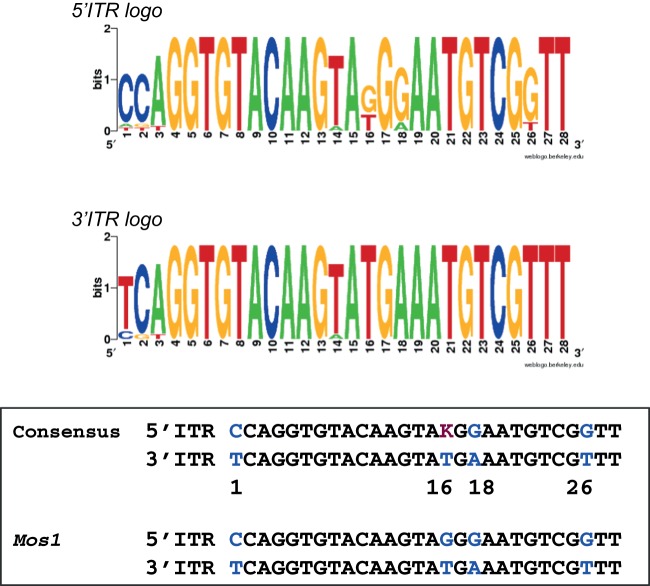
ITR sequence analyses. Sequences of the *Mos1-*tribe ITRs (Tables 1 and 2) were aligned to obtain 5′ and 3′ITRs logos and consensus. The majority rule was used to define variable positions, which are 1, 2, 3, 14, 18, and 26 in the 5′ITR and 1, 2, 3, and 14 in the 3′ITR. Doing so, the 24 conserved positions in *Mos1* ITRs are conserved in the consensus ITRs (black letters). When compared to each other, 5′ and 3′ consensus ITRs contain three clear differences at positions 1, 18, and 26, which are the same as that found in *Mos1* ITRs (blue letters). Position 16 (purple letter) in the 5′ consensus remains ambiguous, being either a G (as in the 5′ *Mos1* ITR) or a T (as in the 3′ *Mos1* ITR).

According to Rouault *et al* [2], the *mariner* family consists of several subfamilies, which are further divided into tribes of elements derived from a common ancestor or founder. In an attempt to reconstruct the sequence of the ancestral *Mos1* ITRs, 5′ and 3′ITR sequences from 14 full-length elements of the *Mos1*-tribe were analyzed (Tables 1 and 2). The *Mos1*-like 5′ITR sequences were found unchanged in nine different copies, whereas the *Mos1*-like 3′ITR sequences were found unchanged in 12 different copies. These observations suggested that *Mos1* could be the *Mos1*-tribe founder element. This was supported by DNA logos and consensus sequences (Figure 6), which demonstrated that the 3′ITR consensus is identical to the *Mos1* 3′ITR, the 3′ITR sequence being highly conserved in the tribe. The 5′ITR consensus is almost identical to *Mos1* 5′ITR, with a single ambiguity. The position 16 is either a T or a G in the consensus, whereas this is a G in the *Mos1* 5′ITR. In a previous study, we analyzed the effect of *Mos1* 5′ and 3′ITRs sequence differences (at positions 1, 16, 18 and 26) on both transposase binding and transposition efficiency [13]. The most important position with regard to the transposase affinity (and subsequent transposition efficiency) was position 16. A *Mos1* 3′ITR mutated at this position had a reduced affinity for the transposase (about 90%), thus resembling a 5′ITR. Mutations at the three other positions had a minor impact on the transposase affinity (≈50%). Although sequence alignments argue for a *Mos1*-tribe founder element having the current *Mos1* ITRs, the variation at position 16 on the 5′ITR could provide a chance (for elements with a T) to be more efficient in transposition and less sensitive to transposase concentration.

**Table 1:** 5′ ITR sequences of the *Mos1*-tribe elements.

5′ITR sequences	Accession number
atAGGTGTACAAGTAtGGAATGTCGGTT	AB091770 (Ds)
CCAGGTGTACAAGTAGGGAATGTCGGTT	AF373028 (Md) AF355143 (Bg) M14653 (Dm) X89926 (Dm) X78907 (Ds) X89923 (Dm) X89924 (Dm) X89925 (Dm) X78906 (Dm)
CCAGGTGTACAAGTAtGGAATGTCGGTT	AF035570 (Dse) X86159 (Zt)
CgtGGTGTACAAGTAtGaAATGTCGTTT	X89927 (Ds)
tCAGGTGTACAAGaAtGaAATGTCGtTT	X86158 (Dt)

The accession numbers are given on the right. The host species is given in brackets. Ds: *Drosophila simulans*; Md: *Musca domestica*; Zt: *Zaprionus tuberculatus*; Dm: *Drosophila mauritiana*; Dse: *Drosophila sechelia*; Dt: *Drosophila tsacasi*; Bg: *Blattella germanica.* The differences between the consensus and the element are in underlined lower cases letters. The Mos1 sequence and accession number are in bold.

**Table 2:** 3′ ITR sequences of the *Mos1*-tribe elements.

3 ITR sequences	Accession number
cgtGGTGTACAAGTATGAAATGTCGTTT	X89927 (Dse)
TCAGGTGTACAAGTATGAAATGTCGTTT	X86158 (Dt) AF373028 (Md) M14653 (Dm) AF355143 (Bg) X89926 (Dm) X78907 (Ds) X89923 (Dm) X89924 (Dm) X89925 (Dm) AB091770 (Ds) X78906 (Dm) AF355143 (Bg)
cCAGGTGTACAAGaATGAAATGTCGTTT	X86159 (Zt)

The accession numbers are given on the right. The host species is given in brackets. Ds: *Drosophila simulans*; Md: *Musca domestica*; Zt: *Zaprionus tuberculatus*; Dm: *Drosophila mauritiana*; Dse: *Drosophila sechelia*; Dt: *Drosophila tsacasi*; Bg: *Blattella germanica.* The differences between the consensus and the element are in underlined lower cases letters. The *Mos1* sequence and accession number are in bold.

## Discussion

*Mos1* displays striking features among the *mariner* family: it is the only *mariner* element for which transposition activity is easily detected in *Drosophila* natural populations (*D. mauritiana*, and *D. simulans*), as well as in lab strains (*D. melanogaster*). In addition, *Mos1* can be conveniently handled for biochemical purposes. In the current work, we provide strong indications that *Mos1* is likely to be the ancestral founder copy of the tribe. Although it is effectively used for transgenesis in invertebrates [25]–[26], *Mos1* has been found ineffective in human cells, in contrast to *Hsmar1* or *Himar1* [9]–[10]. We provide new insights on the intrinsic mechanisms driving the self-regulation of *Mos1*, pinpointing discrete differences between the various members of the *mariner* family. These differences have to be taken into account to better understand the dynamics of *mariner* elements spreading.

Our main finding is that upon *Mos1* transposition, the recruitment of the second ITR occurs cooperatively. Cooperative DNA binding is well known for transcription factors, including cooperative binding to distant DNA binding sites [27]. The specificity of the *Mos1* model is that the cooperativity concerns a transposase dimer already bound to DNA. We assume that after SEC2 assembly, the transposase dimer/ITR complex changes its conformation prior binding the second ITR to form the PEC. Two thin bands corresponding to SEC2 were detected in the figure 3A–B, supporting the occurrence of SEC2s with two different conformations. Our findings argue for a greater affinity between the ITR and the transposase dimer in the re-organized SEC2 than between the ITR and the free transposase dimer. This is in agreement with our knowledge on SEC2 and PEC assembly [7] [28]. Cooperativity would be enhanced by DNA supercoiling, placing the second ITR close to the preformed SEC2. Two studies demonstrate the importance of DNA supercoiling in *mariner* excision [28] and transposition [29]. Cooperativity favours the use of two closely spaced ends. As a consequence, the PEC is formed before the second ITR is saturated by another transposase dimer. The fact that ITR saturation was also not detected in the *Hsmar1* model [8] argues that the cooperative recruitment of the second ITR is a common feature of *mariner* elements transposition. Hence, we confirm that *mariner* transposition is orientated both in space and time: it begins by binding a transposase dimer at one end of the element to form SEC2, and then recruiting the second end to form the PEC. This cooperative assembly of the PEC appears particularly advantageous for *Mos1*-like elements that contain a unique high affinity binding-site (the 3′ITR) for the transposase [13] [30].

We observed that *Mos1* transposition rate increased with the transposase concentration, whatever the pseudo-*Mos1* substrate (with identical ITRs or not). A relationship between MOS1 concentration and transposition regulation was reported for the first time by Hartl and colleagues [12]. Using a genetic approach, they postulated the “overproduction inhibition" (OPI) as a mechanism aimed at down-regulating transposition of *Mos1* when the transposase is over expressed. While they did not measure the actual concentration of the transposase, Hartl and colleagues suggested that its overproduction promotes the formation of high-order oligomers (or aggregates) that were unable to support transposition. This mechanism implied a transposase concentration threshold, above which the whole sample was aggregated and thus inactive. OPI was claimed in the case of *Hsmar1* [8] and *Himar1* [31] *in vitro* transposition assays, while neither OPI nor transposase concentration-dependent aggregation was detected in our assays. It is thus unlikely that OPI is obligatory required for the down-regulation of *mariner* elements transposition. Transposition inhibition relying on transposase concentration can be explained by the physicochemical properties of the transposase, *i.e.* its propensity to aggregate.

Our results argues that *Mos1* intrinsic properties make the element weakly efficient for transposition. The natural ITRs conformation is about 20 times less efficient for transposition than a conformation with two identical 3′ITRs. Even with a pair of efficient 3′ITRs, the *Mos1* transposition rate remains 10 times lower than that of *Hsmar1* assayed under identical conditions (personal data). This shed light onto results obtained in previous *Mos1* transposition assays in eukaryotic cells. Fischer and colleagues have shown that the transposition supported by a pseudo-*Mos1* with the natural ITRs conformation is rather inefficient in human cells [10]. Similar results were obtained in cultured insect cells (*B. mori*) [32]. Moreover, every assay of transgenesis involving *Mos1* was made with a pseudo-*Mos1* containing the natural ITRs conformation. Some were successful [25] but others were not [9]. In the need for improved *Mos1*-based vectors, our results strongly suggest the use of two identical 3′ITRs, together with hyperactive transposases [22].

Biotechnological applications call for very efficient transposition systems, during a short time frame. In fact, the natural *Mos1* element does not meet this criterion. However, beyond biotechnological concerns, our results pave the way for a better understanding of the dynamics of *mariner* transposition in natural populations that concerns long time periods. *Mos1*-related elements have successfully colonized several insect genomes, by the way of recurrent horizontal transfers. The element is still active, and we show that the current *Mos1* ITRs correspond to the ancestral ones. On the one hand, the low transposition efficiency of *Mos1*-related elements has not impaired its spread and persistence within populations. On the other hand, it is consistent with the low copies number of *Mos1*-related elements observed in most host genomes. We speculate that this low efficiency was bypassed at least in part by the self-regulatory mechanisms described here, *i.e.* the cooperative formation of the PEC and the biophysical properties of MOS1 relative to aggregation. It would be of great interest to investigate the dynamics of a *Mos1* element with two 3′ITR upon genome invasion in laboratory strains of drosophila.

Finally, *Mos1* and *Hsmar1*-related elements display differences that are relevant to their dynamics, *i.e*. low copies number but persistence for *Mos1*, and a larger number of *Hsmar1* copies, though dead copies. MOS1 is not sensitive to OPI, in contrast to HsMAR1. MOS1 is less efficient in promoting transposition and *Mos1* contains a single high-affinity binding site for the transposase while *Hsmar1* has two. Although we know that this point of view is simplistic, we believe necessary to explore the intrinsic properties of every *mariner* element to better understand their population dynamics, whether natural or experimental.

## Materials and Methods

### Proteins

The wild type *Mos1* transposase (MOS1: 345 amino acids) was produced and purified as a fusion protein linked to maltose-binding protein (MBP-MOS1), using the pMal-c2 system (New England Biolabs) and following the Manufacturer's instructions. A cleavage site for the factor-Xa protease exists in the fusion between MBP and MOS1.

### EMSA for PEC analyses

The oligonucleotides used in EMSA correspond to the following sequences. The double strand, short pre-cleaved 3′ITR (short-PC) was obtained by annealing the non-transferred strand, 5′-GGTGTACAAGTATGAAATGTCGTTTGATCCCCCGGGCTGCAGG-3′ and the transferred strand, 5′- AATTGCTGCAGCCCGGGGGATCAAACGACATTTCATAC TTGTACACCTGA-3′. The long, double-strand pre-cleaved 3′ITR (long-PC) was obtained by annealing the non-transferred strand, 5′-GGTGTACAAGTATGAAATGTCGTTTCCC TCGAGGTCGACGGTATCGATAAGCTTGATGGCCGC-3′, and the transferred strand, 5′-TCCCGCGGCCATCAAGCTTATCGATACCGTCGACCTCGAGGGAAACGACATTTCATACTTGTACACCTGA-3′.

Classical binding reactions were carried out in 50 mM NaCl, 0.5 mM DTT, 10 mM Tris (pH 9), 7.5% glycerol, and 100 ng of BSA. Each 20 µl of the reaction mixture contained 5 pmol of ^32^P-labeled, short-PC oligonucleotide, and 5 pmol of purified MOS1 in fusion with MBP, unless otherwise stated. Reactions were carried out in 5 mM MgCl_2_ for 30 min at 30°C. The pre-cleaved 3′ITR used was the short form. Reaction products were separated using 6% native polyacrylamide/0.25X TBE gels (30∶0.93 acrylamide-bisacrylamide). Gels were run at 200 V for 3 hours, and then autoradiographed.

Short/long transposase analyses were assembled as indicated for classical binding, using 5 pmol of purified transposase. After incubating for 30 min at 30°C (to allow the complexes to be formed), time-course digests of the complexes by factor-Xa were carried out according to the Manufacturer's instructions (New England Biolabs) (1 µl factor-Xa in 5 mM CaCl_2_) at 25°C for the specified times. Control experiments were performed without factor-Xa. The cleavage of factor-Xa led to the separation of MOS1 and MBP proteins. Reaction products were separated using 6% native polyacrylamide/0.25X TBE gels (30∶0.93 acrylamide-bisacrylamide). Gels were run at 200 V for 3 hours, and then autoradiographed.

Short/long ITR analyses were carried out as indicated for classical binding, using 5 pmol of purified transposase, and a mixture of short-PC and long-PC (final concentration 5 pmol), as specified in the text. Reaction products were separated using 6% native polyacrylamide/0.25X TBE gels (30∶0.93 acrylamide-bisacrylamide). Gels were run at 200 V for 3 hours, and then autoradiographed.

Quantifications were done using the ImageGauge V4.22 software. The graph was draw and the B*max* extrapolated using GraphPad Prism version 4.0c for Macintosh (GraphPad Software, La Jolla California USA, www.graphpad.com).

### *In vitro* transposition assays

*In vitro* transposition reactions were performed using pBC-5T3 or pBC-3T3 (as specified) as both the donor DNA and the target, as previously described [22]. Briefly, the basic transposition reaction mixtures contained 10 mM Tris-HCl (pH 9), 50 mM NaCl, 20 mM MgCl_2_, 0.5 mM EDTA, 5 ng/ml BSA, transposase (purified MBP-MOS1) and donor DNA, in a volume of 20 µl. The concentrations of donor DNA (pBC-3T3 or pBC-5T3) and transposase (purified MBP-MOS1) were as specified in the text or figures legend. The reactions were allowed to proceed for 30 min at 30°C. After phenol-chloroform extraction, and ethanol precipitation, 10% of the reaction mixture was transformed in electro-competent JM109 *E. coli.* Appropriate dilutions of each reaction mixture were plated on LB-tetracycline (12.5 µg/ml) agar and LB-chloramphenicol (80 µg/ml) agar to score the transposition rate. The transposition rate was the number of Tet^R^ colonies divided by the number of Chloram^R^ plus Tet^R^ colonies. Each condition was done at least five times in two independent experiments.

### ITR sequences analyses

5′ and 3′ITR sequences of 14 *Mos1*-related elements were compared for each element. For the analyses, only full-length elements that were not obtained by PCR were retained. In other words, we only retained full-length genomic copies [33]–[36]. DNA logos were done using the web software (http://weblogo.berkeley.edu/logo.cgi) [37].

### Statistical analyses

Statistical analyses were performed using GraphPad Prism version 4.0c for Macintosh (GraphPad Software, La Jolla California USA, www.graphpad.com). We used a Kruskal-Wallis one-way analysis of variance by ranks. This is a non-parametric method for testing whether samples originate from the same distribution. It is used for comparing more than two samples that are not related. The factual null hypothesis is that the populations from which the samples originate have the same median. Analyses were done using an alpha level of 5% (α = 0.05). The calculated Kruskal-Wallis value is then compared to the critical value. When the Kruskal-Wallis test leads to significant results (*i.e.* calculated Kruskal-Wallis value > critical value) then at least one of the samples is different from the other samples. The test does not identify where the differences occur or how many differences actually occur. In order to identify where the differences occur, sample contrasts between individual sample pairs (or post hoc tests) were done. Multiple comparisons were done using the Dunn's test (pairwise comparisons) and the Bonferroni correction to determine if the post-hoc tests are significant.
